# Convolutional Neural Network-Based Models for Near-Infrared Prediction of Nutritional Quality in Multi-Product Animal Feeds

**DOI:** 10.3390/ani16111676

**Published:** 2026-05-30

**Authors:** Xueping Yang, Zhengling Liu, Fuyu Yang, Yanli Lin, Paolo Berzaghi, Salvador Castillo-Girones

**Affiliations:** 1 College of Grassland Science, China Agricultural University, Beijing 100193, China; 2 College of Animal Science, Guizhou University, Guiyang 550025, China; 3 Department of Animal Medicine, Production and Health, University of Padova, 35020 Legnaro, Italy; 4 Cranfield Environment Centre, Cranfield University, Bedfordshire MK43 0AL, UK

**Keywords:** near-infrared spectroscopy, animal feed, convolutional neural network, XGBoost algorithm, Grad-CAM

## Abstract

Near-infrared spectroscopy (NIRS) is widely used for rapid and non-destructive evaluation of feed nutritional quality, but robust calibration remains challenging for heterogeneous multi-product feed datasets. This study evaluated convolutional neural network (CNN)-based models for predicting crude protein (CP) and acid detergent fiber (ADF) using a previously published NIR database containing forage and grain-based feeds. A one-dimensional CNN and two hybrid models, CNN combined with partial least squares regression (CNN+PLS) and XGBoost (CNN+XGBoost), were developed and compared with conventional PLSR calibration models based on either the pooled multi-product dataset or product-specific subsets. Model performance was assessed using an independent internal hold-out test set generated within the same database. For CP prediction, CNN-based models achieved strong performance on the hold-out test set, with testing R^2^ values of 0.98 and RMSEP values of 0.60–0.62, showing a clear reduction in prediction error compared with the global PLSR model. For ADF, CNN and CNN+PLS provided only modest improvements over global PLSR, whereas CNN+XGBoost showed weaker generalization for ADF. Product-wise results further indicated that ADF prediction was more strongly affected by feed matrix and product category than CP prediction. Grad-CAM examples suggested that CNN activation patterns were broadly consistent with known protein- and fiber-related absorption regions, although this interpretation should be regarded as illustrative evidence of spectral coherence rather than direct chemical causality. Overall, CNN-based models, particularly CNN+PLS, showed promise for improving NIRS prediction of CP in heterogeneous feed datasets, while their advantage for ADF was limited. Further validation using independent external datasets and multi-instrument conditions is required before routine implementation.

## Simple Summary

Accurate evaluation of animal feed quality is important for improving livestock nutrition, reducing feed waste, and supporting more efficient animal production. Traditional laboratory testing can be slow, costly, and time-consuming, especially when many different types of feeds need to be analyzed. This study explored whether near-infrared spectroscopy, a rapid and non-destructive light-based method, combined with modern computer models could be used to predict key feed quality traits. The study focused on crude protein, which reflects the protein value of feed, and acid detergent fiber, which is related to the less digestible fiber portion of feed. Different computer models were tested using a large collection of forage and grain-based feed samples. The results showed that these models predicted crude protein very accurately and also provided useful predictions for fiber, although fiber was more affected by differences among feed types. The models also helped identify meaningful wavelength regions linked to protein and fiber information. Overall, this study shows that advanced computer-assisted near-infrared analysis can support faster and more practical feed quality evaluation, which may help farmers, laboratories, and feed companies make better feeding and quality-control decisions.

## 1. Introduction

Near-infrared spectroscopy (NIRS) has become one of the most widely used rapid analytical techniques for evaluating feed nutritional quality because it enables non-destructive and high-throughput prediction of chemical and nutritional constituents. In animal nutrition, NIRS has been applied to estimate crude protein (CP), fiber fractions, dry matter, digestibility-related parameters, and other feed quality traits, thereby supporting feed formulation, quality control, and precision nutrition management [1,2,3,4]. Recent systematic and narrative reviews have further emphasized that NIRS can provide rapid information on livestock diet quality and has practical value for routine nutritional monitoring, although its analytical performance remains highly dependent on calibration robustness and dataset representativeness [3,4].

The development of robust NIRS calibration models is particularly challenging for heterogeneous multi-product feed databases. In such databases, spectral variability is driven not only by chemical composition, but also by botanical origin, processing method, particle size, moisture status, and physical matrix structure. Forage and grain-based feeds differ markedly in chemical composition and spectral background, and even for the same target constituent, the relevant spectral information may be expressed differently across product categories because of differences in matrix composition, scattering behavior, and physical structure. As a result, the relationship between spectra and reference values is often locally structured rather than globally uniform. A calibration model developed using the entire pooled database must therefore simultaneously describe shared chemical signals and product-specific spectral variation. Conventional global partial least squares regression (PLSR) models remain attractive because they provide a single, robust, and easily deployable prediction equation for routine NIRS applications. Previous studies using large multi-product NIRS databases have shown that local or product-aware calibration strategies can improve prediction in some cases, indicating that spectral–chemical relationships may vary across product categories [5,6,7,8,9]. Therefore, in this study, global PLSR was used as a conventional reference model rather than regarded as an inadequate method, and its performance was directly compared with CNN-based models using the same training and hold-out test sets. One possible strategy to reduce matrix heterogeneity is to develop product-specific calibration models. Compared with a single global model, product-specific models may better describe within-product spectral variation because they are trained on more homogeneous subsets. Nevertheless, this strategy also has practical limitations. It requires reliable product classification before prediction, sufficient reference samples for each feed category, and additional model maintenance when new product types or mixed feed matrices are introduced. Therefore, a central methodological question is how a modelling strategy can use the full pooled database while still accounting for product-dependent variation. Conventional linear approaches can address this issue to some extent through global PLSR, product-specific PLSR, local calibration, or other stratified calibration strategies. However, these approaches usually rely on either a single linear latent-variable structure for the whole database or predefined product grouping before prediction. In contrast, CNN-based models may provide an alternative way to learn latent spectral representations directly from the pooled spectra, without explicitly assigning a separate calibration equation to each product category. Thus, the aim of this study was not to assume that PLSR or other linear models cannot address matrix heterogeneity, but to compare whether CNN-based representation learning provides additional predictive or interpretative value relative to conventional PLSR strategies [5,6,7,8,9].

Deep learning provides a potential approach to this problem by learning hierarchical spectral features directly from high-dimensional data. In particular, one-dimensional convolutional neural networks (1D-CNNs) are well suited to NIR spectra because convolutional filters can extract local wavelength patterns and combine them into higher-level representations. Recent studies and re-views have shown that CNNs can be used for end-to-end spectral modelling, feature extraction, dimensionality reduction, and interpretable spectral analysis in food and agricultural applications [10,11,12,13]. Luo et al. systematically summarized CNN applications in spectral analysis and highlighted their roles in end-to-end modelling, spectral dimension reduction, and model interpretability [11]. Kawamura et al. also demonstrated that 1D-CNNs can effectively process continuous visible–near-infrared spectral data for quantitative prediction tasks [12]. In this context, CNNs may offer advantages over purely linear chemometric models by capturing nonlinear spectral patterns and reducing the dependence on manually defined wavelength selection or highly specific preprocessing strategies.

Hybrid modelling strategies may further clarify whether the value of CNNs lies mainly in direct nonlinear regression or in their ability to generate informative latent spectral representations. In such approaches, CNN-extracted features can be used as inputs for downstream regression algorithms, such as PLSR or XGBoost. This strategy combines deep feature extraction with established chemometric or machine-learning regression methods and may be particularly relevant for large and heterogeneous spectral datasets, where both nonlinear feature learning and robust regression are required [10,11,12,13].

The performance of CNN-based NIR models is also expected to be constituent-dependent. CP is mainly associated with N–H and C–H overtone and combination absorptions, which tend to provide relatively distinct and chemically interpretable signals in the NIR region. In contrast, acid detergent fiber (ADF) represents a structurally complex fraction composed mainly of cellulose and lignin, with contributions from broad and overlapping O–H and C–H combination bands. ADF prediction is therefore more susceptible to matrix effects related to forage type, maturity, particle size, and processing history [14,15,16,17]. These differences suggest that CNN-based models may not improve all nutritional traits equally, and that CP and ADF provide a useful contrast for evaluating whether deep spectral representations are more effective for constituents with localized absorption features than for traits dominated by diffuse and overlapping spectral information.

Although prediction accuracy is the primary criterion for evaluating calibration models, interpretability is also important when deep learning models are applied to NIR spectroscopy. Unlike linear chemometric models, CNNs learn latent spectral representations that are not directly linked to individual wavelengths or classical regression coefficients. Therefore, visualization methods such as gradient-weighted class activation mapping (Grad-CAM) can help identify the spectral regions contributing to CNN predictions and assess whether the learned features are consistent with known chemical absorption mechanisms [18]. This step is particularly relevant for heterogeneous multi-product feed datasets, where models may capture product-specific background variation rather than target-related chemical information.

Therefore, the present study re-analysed a previously published heterogeneous multi-product feed NIR database to evaluate the predictive performance, matrix-dependent robustness, and interpretability of CNN-based calibration models for CP and ADF prediction. The purpose of this work was not to introduce a newly generated experimental dataset, but to examine whether CNN-based and hybrid modelling strategies could provide additional value when applied to an existing large multi-product feed database with substantial spectral and compositional heterogeneity. A standard 1D-CNN model and two hybrid models, CNN+PLS and CNN+XGBoost, were developed and compared with conventional PLSR calibration strategies based on either the pooled database or product-specific subsets. In addition, Grad-CAM was used to identify the spectral regions contributing to CNN predictions and to assess whether the learned spectral features were consistent with known NIR absorption features of protein- and fiber-related constituents. Because the hold-out set was generated within the same database, the validation should be interpreted as independent internal hold-out validation rather than true external validation. The specific objectives were to: (1) determine whether CNN-based models improve CP and ADF prediction compared with conventional PLSR calibration strategies; (2) examine whether CNN-derived features benefit downstream PLSR and XGBoost regression; (3) assess the product-wise behavior and matrix dependent robustness of CNN-based models across different feed matrices; and (4) evaluate the spectral coherence of CNN-focused wavelength regions using Grad-CAM.

## 2. Materials and Methods

### 2.1. Dataset and Spectral Acquisition

This study used a previously published near-infrared spectroscopy (NIRS) multi-product feed dataset [5,6]. The dataset comprised 3222 samples representative of both forage and grain-based animal feeds. Seven feed product categories were included: four forage products, namely hay (HAY, *n* = 1211), corn silage (CSL, *n* = 471), small grain silage (SGS, *n* = 276), and total mixed ration (TMR, *n* = 278), and three grain-based products, namely corn (*n* = 478), oat (*n* = 161), and wheat (*n* = 347). These samples represented common feed types used in ruminant nutrition and covered a broad range of compositional and spectral variability. Descriptive statistics for crude protein (CP) and acid detergent fiber (ADF) by product category have been reported previously [5,6]. To ensure comparability with previous calibration studies, the same training and testing subsets were used in the present work. Because reference values were not available for all target constituents in all samples, trait-specific modelling datasets were used. After removing samples without the corresponding reference values, 3214 samples were available for CP modelling and 3151 samples were available for ADF modelling. The exact numbers of samples assigned to the training and hold-out test sets for each product category are provided in Supplementary Table S1. The Kennard–Stone algorithm was applied within each product category to generate the training and hold-out test sets for each trait-specific dataset. For CP, 2891 samples were used for training and 323 samples for testing. For ADF, 2836 samples were used for training and 315 samples for testing. The same trait-specific training and hold-out test sets were used for all models within each target constituent. Spectral measurements were collected using a Foss NIRSystems 5000 scanning monochromator (Foss Analytical A/S, Hillerød, Denmark). Spectra were recorded over the wavelength range of 1100–2498 nm at 2 nm intervals. Before spectral acquisition, samples were dried either using a microwave system or in a forced-air oven at 60 °C and then ground to pass through a 1 mm sieve. Spectra were acquired in reflectance mode using 55 mm diameter circular sample cups and were expressed as absorbance spectra for subsequent analysis. This was a secondary modelling study based on a previously published feed NIR database. No new feed samples, chemicals, reagents, commercial cell lines, or biological materials were purchased or used in the present study.

### 2.2. Reference Chemical Analyses

In the original database, reference chemical analyses were conducted to determine crude protein (CP) and acid detergent fiber (ADF) concentrations. CP was determined using the Kjeldahl method, with nitrogen concentration converted to crude protein using a factor of 6.25. ADF was analyzed according to standardized fiber analysis procedures. Both CP and ADF values were expressed as percentage of dry matter (% DM).

### 2.3. Spectral Preprocessing

All spectral preprocessing and model development were performed in R version 4.2.1 [19] and Python 3.11. To avoid model-dependent preprocessing selection, all models reported in this study were developed using the same predefined preprocessing pipeline. Raw reflectance spectra were first corrected using standard normal variate followed by detrending (SNVD) to reduce multiplicative scattering effects, baseline shifts, and physical variation associated with particle size and sample presentation. The SNVD-preprocessed spectra were then subjected to Savitzky–Golay smoothing and first-order derivative transformation to reduce spectral noise and enhance overlapping absorption features. For the Savitzky–Golay transformation, a second-order polynomial, a window size of seven wavelength points, and a first-order derivative were used. Because the Savitzky–Golay transformation was applied without edge padding or extrapolation, wavelength variables affected by the filter window at both spectral edges were removed. With a window size of seven wavelength points, three wavelength variables were removed from each end of the spectra. As a result, the final preprocessed spectra covered 1106–2492 nm and contained 694 wavelength variables. The final spectral input used for conventional PLSR, CNN, CNN+PLS, and CNN+XGBoost models was therefore SNVD followed by Savitzky–Golay smoothing and first-order derivative transformation. No model-specific preprocessing selection was performed, ensuring that differences in model performance reflected the modelling strategies rather than different spectral preprocessing choices. The preprocessing procedures were performed using functions from the ‘prospectr’ version 0.2.7 [20]. Model performance was evaluated using the coefficient of determination (R^2^), mean absolute error (MAE), root mean square error (RMSE), and root mean square error of prediction (RMSEP) and ratio of performance to deviation (RPD). RMSE was used to describe calibration error in the training set, whereas RMSEP was used to describe prediction error in the independent internal hold-out test set. RPD was calculated as the standard deviation of the reference values in the hold-out test set divided by RMSEP.

### 2.4. Modeling Approaches

#### 2.4.1. Conventional PLSR Calibration Models

Partial least squares regression (PLSR) was used to establish conventional calibration models. PLSR models were developed using the ‘mdatools’ version 0.14.2 [21]. Spectral data were mean-centered and standardized before model development. The optimal number of latent variables was selected according to the first local minimum of the root mean square error of cross validation (RMSECV). The training and test sets were strictly independent, loaded from separate files with no overlap at any stage of the modelling process. Latent variable selection was therefore based solely on training set information, and the test set was reserved exclusively for final internal hold-out evaluation. Two conventional PLSR calibration strategies were considered: the global PLSR model developed using the entire pooled multi-product database was referred to as Global PLSR (GCM) and the product-specific PLSR strategy, in which separate PLSR models were developed for each individual product category, was referred to as Product-specific PLSR (GCP). These two PLSR-based strategies were used as conventional chemometric reference models for evaluating the performance of CNN-based models.

#### 2.4.2. CNN-Based Models

Three convolutional neural network (CNN)-based approaches were developed for predicting CP and ADF: (i) a standard one-dimensional CNN (1D-CNN), (ii) CNN combined with partial least squares regression (CNN+PLS), and (iii) CNN combined with XGBoost (version 3.2) regression (CNN+XGBoost). The input to all CNN-based models consisted of the preprocessed one-dimensional spectral data. Separate CNN models were independently trained for CP and ADF prediction. The same architecture was used for both constituents, but model weights were optimized independently for each target variable.

**Figure 1:**
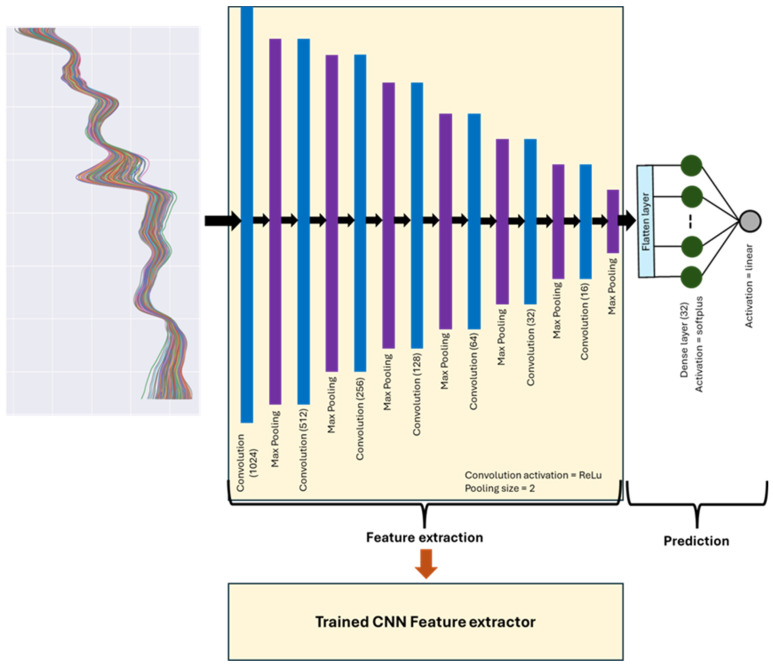
Architecture of the one-dimensional convolutional neural network (1D-CNN) used for direct regression and feature extraction. The model consisted of sequential convolutional and max-pooling layers followed by a dense layer and a linear regression output.

The CNN architecture (Figure 1) was implemented in TensorFlow version 2.10 [22]. The architecture was selected by grid search in Python using 10-fold cross validation on the training dataset, evaluating between 3 and 10 convolutional blocks and testing filter sizes of 2048, 1024, 512, and 256 for the first layer, with each subsequent layer reducing the number of filters by half. The parameter selection criterion was the lowest root mean square error (RMSE). The independent internal hold-out test set was not used at any stage of architecture selection or hyperparameter optimization. The selected architecture consisted of seven sequential one-dimensional convolutional layers with decreasing filter sizes (1024, 512, 256, 128, 64, 32, and 16) and kernel sizes of 3, respectively. Each convolutional layer used ReLU activation and was followed by max-pooling with a pooling window of 2. The convolutional-pooling stack was followed by a Flatten layer, a fully connected Dense layer with 64 neurons and softplus activation, and a final fully connected layer with 32 neurons and a linear output layer for regression, with a dropout of 0.2. The model input consisted of spectra with 694 wavelengths. Early stopping was applied with a patience of 50 epochs using a dedicated validation set constructed from the training data. A fixed random seed was used to ensure reproducibility, adopting the default seed-handling mechanism provided by the library where applicable. This approach ensures that model initialization, data splitting, and any stochastic training procedures can be reproduced consistently across runs.

The model was compiled using mean squared error (MSE) as the loss function and the Adam optimizer with its default learning rate (0.001). Training was run for a maximum of 2000 epochs with a batch size of 100 and data shuffling enabled at each epoch. A validation set was monitored throughout training, and early stopping was applied with a patience of 50 epochs to avoid overfitting based on the MSE of the validation set, ensuring that training halted when no further improvement was observed. Training progress was logged using TensorBoard to monitor training and validation loss curves and confirm the absence of divergent overfitting. The best model was defined as the model state at the epoch with the lowest validation MSE. Model weights were initialised using TensorFlow default (Glorot uniform) initialisation with a fixed random seed to ensure reproducibility.

**Figure 2:**
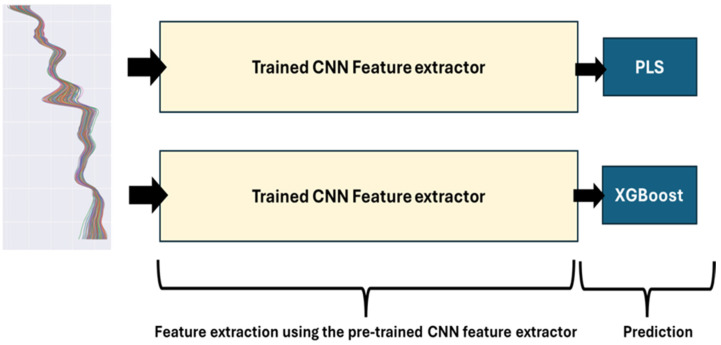
Workflow of the hybrid CNN-based models. CNN-derived latent spectral features were extracted from the trained convolutional-pooling layers and then used as inputs for downstream PLSR and XGBoost regression models.

For the hybrid CNN+PLS and CNN+XGBoost models, the trained CNN was reused as a fixed feature extractor with all convolutional and pooling weights frozen (Figure 2). The Dense and output layers were removed, and each input spectrum was propagated through the retained convolutional-pooling layers. This procedure followed the workflow illustrated in a publicly available example and was adapted here to the present spectral regression task [23,24].

The extracted feature vector used for downstream modelling was taken from the first fully connected layer and consisted of 64 values. Feature vectors were standardised (zero mean, unit variance) prior to downstream regression to avoid scale-related bias. Because the CNN weights were frozen before feature extraction, and because the test set was withheld from all optimization steps, data leakage between training and test sets was avoided.

For CNN+PLS, the number of latent components was optimized by 10-fold cross-validation within the training set using the CNN-derived feature vectors as predictor variables, implemented via scikit-learn version 1.8.0. Although 19 and 21 latent components were selected for ADF and CP, respectively, these were extracted from the CNN-derived latent feature space rather than from the original wavelength variables. To justify the final number of components, RMSECV curves were generated by re-importing the trained CNN models, re-extracting the features, and re-running PLS on the extracted feature vectors. For CNN+XGBoost, the XGBoost algorithm was used [23]. Hyperparameter optimization was performed using grid search with 10-fold cross validation within the training set, evaluating learning rate, maximum tree depth, number of estimators, subsampling ratio, column subsampling ratio, gamma, and minimum child weight. The optimal hyperparameter combination for each target trait was selected based on the lowest cross validation RMSE. The independent internal hold-out test set was not used at any stage of CNN architecture selection, feature extractor training, or downstream regressor optimization. The final selected hyperparameters are summarized in Table 1. The use of 1D-CNNs as spectral feature learners is also consistent with recent NIR applications in feed-protein prediction, where CNN-based models have been used to extract informative spectral features from feed spectra [25].

#### 2.4.3. Model Performance Evaluation

The predictive performance of the developed models was evaluated using specific statistical metrics to quantify accuracy and data spread. The coefficient of determination (R^2^) measured the proportion of variance in the reference data successfully explained by the model. General prediction errors were quantified using the mean absolute error (MAE) for average absolute differences, while the root mean square error (RMSE) was used to penalize larger prediction deviations [26,27].

To evaluate systematic errors, the slope of the regression line and the mean bias were calculated to determine proportional agreement and average overestimation or underestimation, respectively. The standard error of prediction (SEP) was computed to measure the precision and variance of the prediction errors specifically within the independent internal hold-out test set. Finally, the ratio of performance to deviation (RPD) was utilized to assess the practical utility of the models, with higher values indicating superior predictive robustness [26,28].

### 2.5. Gradient-Weighted Class Activation Mapping (Grad-CAM)

To enhance the interpretability of the CNN-based models, Gradient-weighted Class Activation Mapping (Grad-CAM) was employed as a visualization technique to identify the spectral regions most influential in the model’s predictions [18]. Since CP and ADF are continuous variables, Grad-CAM was adapted to a regression context: rather than computing gradients with respect to a class score, gradients were computed with respect to the scalar regression output of the model for each target constituent.

Grad-CAM was applied to the trained CNN models following four steps: (1) Gradient computation: for each input spectrum, a gradient model was constructed using TensorFlow by connecting the model inputs to both the output of the target convolutional layer, which was the last convolutional layer, and the final scalar regression output. Gradients of the predicted value with respect to the feature maps were computed using automatic differentiation via tf.GradientTape; (2) Gradient pooling: the gradients were averaged across the spectral dimension of the feature maps to produce a weight vector reflecting the relative importance of each filter; (3) Heatmap generation: the weighted sum of the convolutional feature maps was computed, ReLU was applied to retain only positively contributing activations, and the resulting heatmap was normalized to the range of 0–1. The heatmap was then linearly interpolated to the original input wavelength scale and overlaid onto the original NIR spectrum for visualization; (4) Interpretation: the rescaled heatmap was overlaid onto the original NIR spectrum and compared with known NIR absorption bands for CP and ADF to assess whether the model activation patterns were spectrally coherent with protein- and fiber-related absorption features.

This method allowed a visual assessment of the model’s decision-making process, particularly in relation to protein and fiber absorption, and helped evaluate whether the CNN activation patterns were consistent with known NIR absorption regions, without implying direct chemical causality.

## 3. Results

### 3.1. Spectral Overview of the Pooled Multi-Product Dataset

A detailed vibrational assignment of this near-infrared spectroscopy database has been reported previously for multi-product feed calibrations [5,6]. Therefore, this section focuses on the spectral structure revealed by the standard normal variate-pretreated heatmap of the pooled dataset rather than repeating a detailed band-by-band interpretation. Each row in the heatmap represents one feed sample, and each column corresponds to wavelengths from 1100 to 2498 nm. Within each product category, samples were ordered by increasing crude protein content to visualize compositional gradients across the pooled database. The raw absorbance spectra for each product category are provided in Supplementary Figure S1.

**Figure 3:**
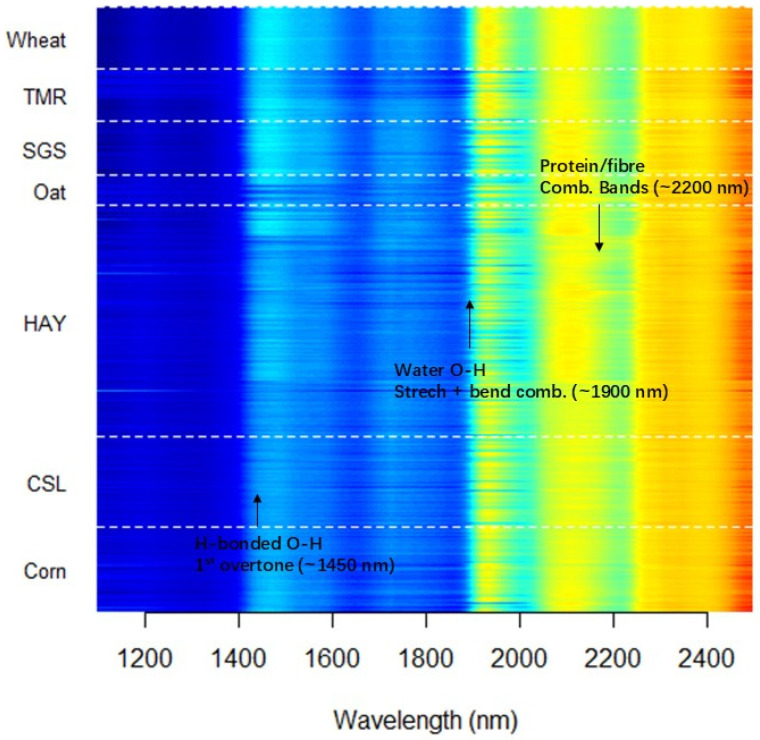
Exploratory heatmap of preprocessed near-infrared spectra from the pooled multi-product feed dataset. Samples within each product category were ordered by increasing crude protein content. SGS, small grain silage; CSL, corn silage; TMR, total mixed ration.

The heatmap revealed clear spectral heterogeneity among the seven feed product categories (Figure 3). Therefore, Figure 3 should be interpreted as an exploratory visualization of spectral heterogeneity across product categories. The most pronounced spectral variations occurred around approximately 1450, 1900, and 2100–2300 nm, corresponding to typical O–H, N–H, and C–H overtone and combination absorption regions [14,17]. A distinct block structure was observed between forage products, including hay, corn silage, small grain silage, and total mixed ration, and grain-based products, including corn, oat, and wheat. Forage samples showed broader and more heterogeneous absorption patterns, particularly in water- and fiber-associated regions, whereas grain samples displayed more compact and smoother spectral profiles, probably reflecting their relatively starch-rich and compositionally homogeneous matrices.

Ordering samples by crude protein content further revealed within-product spectral gradients. In hay and total mixed ration, increasing crude protein content was accompanied by systematic spectral changes not only in protein-related regions but also in water- and carbohydrate-associated bands. This suggests that protein variation was partly coupled with moisture-related and structural carbohydrate signals. In contrast, crude protein-related gradients in grain products were less pronounced and appeared superimposed on a more homogeneous starch-dominated spectral background. These patterns indicate that the spectral–property relationship in the pooled dataset was strongly influenced by both product-specific variation and cross-product heterogeneity, supporting the need for modelling strategies capable of handling complex multi-product feed spectra.

### 3.2. Overall Model Performance for CP and ADF

Table 2 and Table 3 report the calibration and independent internal hold-out validation statistics respectively for all modelling strategies applied to CP and ADF on the pooled multi-product dataset, using the training–test split described in Section 2. All models were built using the same spectral information and reference data. Therefore, differences in performance reflected how effectively each modelling approach captured informative spectral features from the heterogeneous feed spectra.

For CP prediction, the global PLS model based on the entire multi-product database (GCM) provided the conventional baseline, with an RMSEP of 0.73 and an MAE of 0.55 on the independent internal hold-out set (R^2^ = 0.98). The product-specific PLS calibration (GCP) yielded a marginally higher training R^2^ of 0.98 and a slightly higher RMSEP of 0.77, with an MAE of 0.56, indicating that product-specific stratification did not improve hold-out prediction performance for CP in this dataset. The CNN-based approaches reduced prediction error while maintaining the same testing R^2^. The standard 1D-CNN achieved an RMSEP of 0.62, whereas the two hybrid models, CNN+PLS and CNN+XGBoost, further reduced the RMSEP to 0.60. Compared with Global PLSR (GCM), the best CNN-based models reduced the RMSEP for CP from 0.73 to 0.60 on the independent internal hold-out test set, corresponding to an absolute reduction of 0.13 percentage units and a relative reduction of approximately 18%. Because no bootstrap confidence intervals or repeated train/test splits were available, this improvement should be interpreted descriptively rather than as a formal statistical comparison.

For ADF prediction, all models showed high R^2^ values on the independent internal hold-out test set, but the differences in the RMSEP indicated that model performance should be interpreted cautiously. The GCM baseline yielded an RMSEP of 1.44 and an MAE of 1.05. The GCP model achieved a slightly lower RMSEP of 1.42 and an MAE of 1.03. The standard CNN and CNN+PLS models provided only modest additional reductions in RMSEP, reaching 1.38 and 1.39, respectively. Thus, unlike CP, the improvement obtained by CNN-based modelling for ADF was limited in absolute terms. In contrast, CNN+XGBoost showed poorer generalisation for ADF, with an RMSEP of 1.78 and an MAE of 1.62, which was worse than both GCM and GCP. These results indicate that XGBoost regression head did not provide a benefit for ADF when applied to CNN-derived spectral features in the present dataset [11].

Overall, the pooled validation results showed a constituent-dependent pattern. For both CP and ADF, GCP offered only marginal differences relative to GCM, suggesting that product-specific PLS stratification provided limited added value when models were evaluated on the full heterogeneous test set. CNN-based models provided a clearer improvement for CP than for ADF. Among the CNN-based approaches, CNN+PLS showed stable performance for both constituents, whereas CNN+XGBoost performed well for CP but not for ADF.

### 3.3. Product-Wise Performance of CNN-Based Models

#### 3.3.1. Product-Specific Performance for ADF

Table 4 presents the calibration metrics for ADF prediction models utilizing the training dataset. Across the evaluated architectures, the Product-specific PLSR models consistently demonstrated the most robust calibration performance across all agricultural products, achieving R^2^ values ranging from 0.93 for SGS to 0.98 for both TMR and Oat. In comparison, the Global PLSR strategy yielded slightly lower, R^2^ values between 0.84 and 0.94, accompanied by generally higher error margins. The deep learning approaches, comprising the base CNN, CNN+PLS, and CNN+XGBoost models, exhibited substantial product-dependent variability during training. While these neural network-based models achieved exceptional calibration for specific products, such as TMR with an R^2^ up to 0.99 and Hay reaching up to 0.94, they struggled to fit the data for Wheat, with low R^2^ values ranging from 0.35 to 0.54.

When evaluated on the independent test set (Table 5), the true predictive capabilities of the models were assessed. The Product-specific PLSR maintained superior predictive accuracy, evidenced by high R^2^ scores such as 0.98 for TMR and 0.96 for Corn, and excellent RPD values peaking at 5.00 for Wheat. The CNN and its hybrid variants (CNN+PLS and CNN+XGBoost) demonstrated competitive, though somewhat inconsistent, predictive performance. For instance, the standalone CNN model generated strong predictions for TMR (R^2^ = 0.97, RMSEP = 2.05) and Hay (R^2^ = 0.92, RMSEP = 1.40), but experienced noticeable performance degradation when applied to products like Oat and SGS. Reassuringly, across the majority of the models and products, Bias values remained relatively close to zero and Slope values consistently hovered near 1.0 (ranging predominantly between 0.89 and 0.99), demonstrating an overall absence of severe systematic over or under estimation in the predictions.

A particularly notable observation in the test results is the performance of the CNN-based architectures when predicting ADF in Wheat. Specifically, the standalone CNN yielded an R^2^ of just 0.39, while the CNN+PLS model declined to 0.28. However, it is essential to clarify that product-wise R^2^ can be highly sensitive to the reference-value range and the small size of the hold-out test set. Therefore, a low product-wise R^2^ may occur even when the absolute prediction error is not large. This is illustrated in the Wheat predictions: despite the poor R^2^ values for the CNN models, their absolute error metrics are among the lowest in the dataset [29,30]. For the standalone CNN predicting Wheat, the MAE is merely 0.44 and the RMSEP is 0.59. These errors are quantitatively superior to those seen in products that achieved much higher R^2^ values, such as CSL (R^2^_TST_ = 0.88, MAE = 0.86) or Hay (R^2^_TST_ = 0.92, MAE = 1.09). The narrow range of natural ADF variation in the Wheat samples likely compresses the data’s variance, artificially deflating the R^2^ metric even though the model is generating predictions that tightly match the true chemical values.

Overall, the product-wise ADF results confirmed that model performance was strongly matrix-dependent. Product-specific PLSR generally provided the lowest RMSEP across most feed categories, whereas CNN-based models showed inconsistent product-wise performance.

#### 3.3.2. Product-Specific Performance for CP

Table 6 details the calibration metrics for CP prediction models using the training dataset. Unlike the previous ADF predictions, the deep learning architectures demonstrated exceptional calibration capability for CP across almost all products. The standalone CNN, CNN+PLS, and CNN+XGBoost models consistently achieved R^2^ values exceeding 0.93, with many products such as TMR reaching near perfect fits (R^2^ = 1.00) and Wheat achieving 0.98 across the base CNN and CNN+PLS architectures. These deep learning models yielded low calibration errors, such as an MAE of 0.27 and RMSE of 0.36 for Corn in the standalone CNN model. Among the traditional chemometric approaches, the Product-specific PLSR closely trailed the deep learning models with R^2^ values ranging from 0.93 for Oat to 1.00 for TMR. The Global PLSR, while slightly less accurate than the product-specific and neural network models, still provided strong calibration fits with R^2^ values between 0.85 and 0.93.

The evaluation on the independent test set (Table 7) corroborates the high efficacy of the developed models for CP prediction. Among the CNN-based models, CNN+PLS showed the most favorable product-wise CP prediction performance. It achieved R^2^ values ranging from 0.92 for Oat up to 0.99 for TMR, paired with RPD values peaking at 5.68 for TMR and 5.07 for SGS. The standalone CNN and the Product-specific PLSR also showed good product-wise prediction performance, both achieving an R^2^ of 0.98 for Wheat and 0.99 for TMR, with RPD values well above the threshold typically required for quantitative analysis. The CNN+XGBoost model showed slightly higher error margins and lower RPD values (e.g., RPD of 3.00 for Wheat) compared to the CNN+PLS approach. Across all architectures evaluated in the test set, Bias values remained close to zero and slope values were generally close to 1.0, indicating no major systematic over or under estimation. Overall, CNN+PLS provided a stable and accurate framework for CP prediction across the evaluated feed products.

The superior performance of the CNN+PLS model likely reflects the complementary strengths of both approaches: the CNN can extract complex and informative spectral features, while PLS provides a robust regression framework that is well suited to collinear spectroscopic data and limited sample sizes. This combination can improve prediction stability and reduce overfitting, leading to better overall CP prediction performance [31].

### 3.4. Interpretability of CNN Models Using Grad-CAM

**Figure 4:**
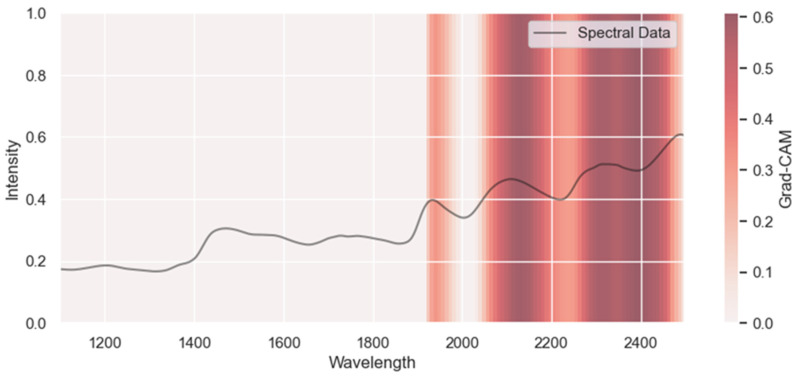
Representative Grad-CAM heatmap for CP prediction based on one randomly selected sample from the independent internal hold-out test set. The activation map was normalized and interpolated to the original wavelength scale, with higher color intensity indicating stronger model activation. This figure is provided as an illustrative individual-sample example and should not be interpreted as an averaged or product-specific heatmap.

**Figure 5:**
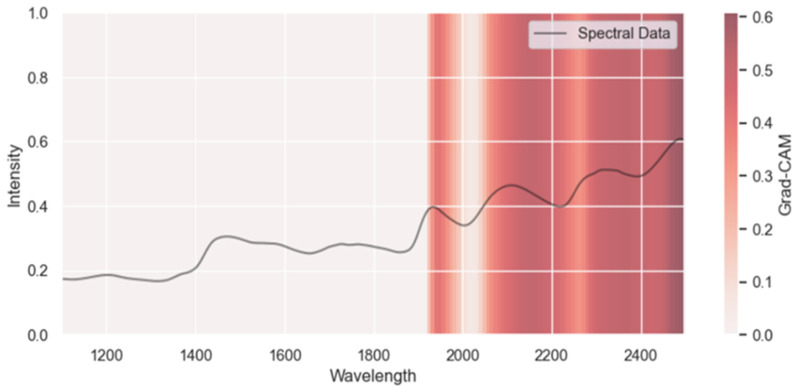
Representative Grad-CAM heatmap for ADF prediction based on one randomly selected sample from the independent internal hold-out test set. The activation map was normalized and interpolated to the original wavelength scale, with higher color intensity indicating stronger model activation. This figure is provided as an illustrative individual sample example and should not be interpreted as an averaged or product-specific heatmap.

Grad-CAM was used to explore whether the CNN models focused on spectral regions consistent with known NIR absorption features for CP and ADF prediction. It was applied to two randomly selected individual samples from the independent hold-out test set, one for CP and one for ADF. Results are shown in Figure 4 and Figure 5, respectively, and should be interpreted as illustrative individual examples rather than as aggregated or product-representative outputs.

For CP prediction (Figure 4), the strongest activations were mainly observed around 1500–1600 nm and 2000–2100 nm. These regions are associated with N–H and C–H overtone and combination bands and are commonly related to protein absorption in near-infrared spectra [14,32]. The activation pattern therefore indicates that the CNN model captured relevant protein-related spectral information rather than arbitrary spectral variation. This interpretation is consistent with previous NIRS studies on cereal and grain protein prediction, in which similar wavelength regions contributed substantially to protein estimation [33,34].

For ADF prediction (Figure 5), the Grad-CAM heatmap showed strong activation around 2000–2100 nm and sustained responses across the broader 2000–2400 nm region. This region is mainly associated with C–H and O–H combination bands from structural carbohydrates, including cellulose, hemicellulose, and lignin. Previous studies have shown that long-wavelength SWIR regions are informative for fiber-related traits in forage and plant materials [15,16,35]. The broader activation pattern observed for ADF is chemically reasonable because ADF is composed of multiple structural components with overlapping absorption features. This interpretation is further supported by NIR band-assignment studies of lignocellulosic materials, which reported that cellulose, hemicellulose, and lignin generate multiple overlapping absorption bands in the 2000–2400 nm region [17].

Compared with CP, ADF showed a broader and less localized activation pattern, suggesting that ADF prediction required the integration of spectral information across a wider wavelength interval. This difference helps explain the model performance observed in this study: CNN-based models performed competitively for CP because protein-related features were relatively localized and specific, whereas ADF remained more challenging due to broader, overlapping, and matrix dependent absorptions. Overall, the Grad-CAM results support the spectral coherence of the CNN models by showing that high-activation regions broadly overlapped with known NIR absorption regions. However, these heatmaps should be interpreted as model-interpretation tools and do not provide direct evidence of chemical causality. The use of Grad-CAM also improves model transparency by linking CNN predictions to known spectral absorption mechanisms [18].

Product-wise Grad-CAM analysis or explicit comparisons between forage and grain matrices may provide additional insight into matrix-dependent model behaviour and should be considered in future studies.

## 4. Discussion

The present study demonstrated that CNN-based models, particularly CNN and CNN+PLS, improved CP prediction on an independent internal hold-out test set derived from a heterogeneous multi-product feed dataset. However, the advantage of CNN-based modelling was not uniform across target constituents. For ADF, CNN and CNN+PLS provided only modest reductions in RMSEP compared with Global PLSR, whereas CNN+XGBoost showed poorer generalization. These results indicate that CNN-based modelling may be more promising for constituents with relatively distinct spectral features, such as CP, than for complex fiber-related traits such as ADF. The results of the present study provide empirical evidence for this methodological consideration. For CP prediction, the global PLSR model achieved an RMSEP of 0.73 on the hold-out test set, whereas CNN, CNN+PLS, and CNN+XGBoost reduced RMSEP to 0.62, 0.60, and 0.60, respectively. This corresponds to an absolute reduction of 0.11–0.13 percentage units and a relative reduction of approximately 15–18% compared with the global PLSR model. However, this advantage was constituent-dependent. For ADF, CNN and CNN+PLS only slightly reduced RMSEP compared with global PLSR, from 1.44 to 1.38 and 1.39, respectively, whereas CNN+XGBoost increased RMSEP to 1.78. These results indicate that the limitation of a single global linear model should not be interpreted as a universal failure of PLSR, but rather as a dataset and constituent dependent issue in heterogeneous multi-product calibration.

The distinct prediction performance between constituents is chemically reasonable, as CP is associated with distinct N-H and C-H overtone bands, whereas ADF represents a structurally complex fraction with overlapping absorptions from cellulose, hemicellulose, and lignin [16,17]. This was reflected in the product-wise results: CP prediction was relatively stable across feed categories (Table 6 and Table 7), whereas ADF showed stronger product-dependent variation (Table 4 and Table 5), with GCP achieving the lowest RMSEP in most individual products. These findings indicate that ADF prediction was more matrix-dependent than CP prediction. The gradient-weighted class activation mapping (Grad-CAM) results supported this, showing strong activation in established protein regions around 1500–1600 nm and 2000–2100 nm for CP. In contrast, ADF prediction required integrating diffuse and matrix-dependent information across the broader 2000–2400 nm region, which explains the modest improvement of CNN-based models over conventional linear models [18].

The hybrid CNN+PLS strategy was promising because it combined automatic feature learning with a regression method well suited to highly collinear spectral variables. Because CNN+PLS matched or slightly improved upon the standard CNN in several comparisons, the results suggest that CNN-derived latent spectral representations may provide useful information for downstream regression. However, this interpretation should be considered preliminary because repeated train/test splits and independent external validation were not available. In contrast, CNN+XGBoost showed poorer generalization for ADF than Global PLSR and CNN+PLS. This may reflect the difficulty of using a tree-based regression head on CNN-derived features for a target trait dominated by broad, overlapping, and matrix-dependent fiber absorptions [11]. Because no direct overfitting diagnostics, repeated resampling, or independent external validation was available, this explanation should be regarded as a plausible interpretation rather than definitive evidence of overfitting. However, the relatively high number of PLS components selected for CNN+PLS indicates a possible risk of overfitting, even though component selection was performed within the training set. Therefore, the stability of the CNN+PLS approach should be further evaluated using repeated resampling and independent external validation in future studies. These findings align with recent perspectives highlighting deep learning as a promising direction for handling nonlinear relationships and matrix heterogeneity in near-infrared modelling [10,11]. Prior reviews have emphasized the value of 1D-CNN models for end-to-end prediction, effective feature extraction, and interpretable spectral analysis in food quality evaluation [11,14]. Furthermore, the successful application of 1D-CNNs to continuous spectral data confirms their utility for quantitative regression tasks in agricultural domains [12].

A major novelty of this work was the large-scale evaluation of standard and hybrid CNN models on a multi-product feed database, combined with Grad-CAM interpretation to link model attention with plausible spectral absorption regions. Despite these advances, several limitations should be acknowledged. First, the validation used in this study was based on an independent internal hold-out test set generated from the same previously published database and should not be interpreted as true external validation. Second, no independent external database, temporal validation set, leave-one-product-out validation, repeated train/test resampling, or bootstrap confidence intervals were available in the present analysis; therefore, small differences in RMSEP among models should be interpreted cautiously. Third, all spectra were acquired using a single Foss NIRSystems 5000 instrument under controlled laboratory conditions, and model transferability to other NIR instruments, laboratories, or field conditions remains untested. Fourth, product categories were imbalanced, ranging from 1206 CP-available HAY samples to 161 OAT samples, which may influence the interpretation of product-wise and pooled validation metrics. Finally, only CP and ADF were evaluated, meaning that the conclusions cannot yet be extended to other nutritional parameters such as NDF, digestibility, or energy without further validation. Therefore, the results should be considered as evidence from an internal validation study rather than proof of routine industrial robustness.

The rationale for using hybrid CNN-based models was to evaluate whether CNN-derived latent spectral features could improve downstream regression compared with direct CNN regression. In the CNN+PLS model, the convolutional layers were used to extract latent spectral representations, while PLSR was used as a regression method suited to collinear spectral features. The results showed that CNN+PLS performed similarly to or slightly better than the standard CNN for CP and ADF, suggesting that CNN-derived features retained useful predictive information for linear regression. In contrast, CNN+XGBoost performed well for CP in the pooled evaluation but showed poorer generalization for ADF and less stable product-wise performance. These findings indicate that the benefit of hybrid modelling depended on both the target constituent and the downstream regression algorithm. Similar matrix-dependent behaviour has also been reported in pasture grass crude protein estimation using machine learning and hyperspectral data [36]. For instance, in a recent assessment of corn feed protein, a 1D-CNN significantly improved predictions over conventional PLSR [24]. Similarly, the 1D-CNN and CNN+PLS architectures in this study reduced the CP prediction error by approximately 18% compared to the global PLS model (RMSEP: 0.60 vs. 0.73). However, the benefit of CNNs appears highly dependent on the target matrix. In predicting complex structural traits like soil carbon and nitrogen, some authors have noted that CNNs require large, comprehensive datasets to definitively outperform PLS [37]. In starch moisture prediction, PLSR with optimal preprocessing was also shown to match or exceed 1D-CNN performance [38]. Recent applications of NIRS combined with deep learning and machine learning for oat hay quality evaluation further support the potential of these methods for forage-related matrices [39]. This mirrors the findings for ADF, where the complex, overlapping signals of structural carbohydrates (cellulose, lignin) meant that CNN+PLS provided only a minor improvement (RMSEP 1.39 vs. 1.44) and the CNN+XGBoost showed poorer generalization, suggesting that the tree-based regression head may be less suitable for CNN-derived features in ADF prediction, reinforcing the necessity of matching the regression algorithm to the complexity of the analytical target.

Future studies should further assess the robustness of CNN-based models using repeated train/test resampling, leave-one-product-out validation, product-balanced sampling strategies, instrument-transfer experiments, and independent external datasets to evaluate whether CNN-derived spectral features remain stable across laboratories, instruments, years, and previously unseen feed matrices.

## 5. Conclusions

This study evaluated CNN-based and hybrid models for NIRS prediction of CP and ADF using a previously published heterogeneous multi-product feed database. CNN and CNN+PLS were promising for CP prediction, reducing RMSEP compared with Global PLSR on the independent internal hold-out test set. In contrast, the advantage of CNN-based models for ADF was limited. CNN and CNN+PLS provided only modest improvements, while CNN+XGBoost showed poorer generalization for ADF. Product-wise evaluation further showed that pooled performance metrics can mask important matrix-dependent differences among feed categories, as illustrated by the low R^2^_TST_ observed for Wheat ADF despite moderate RMSEP values.

Representative Grad-CAM examples suggested that CNN activation patterns were broadly consistent with known protein- and fiber-related NIR absorption regions, but these results should be interpreted as spectral coherence rather than direct chemical causality. Overall, CNN-based models may provide a useful complementary approach for NIRS feed analysis, particularly for CP prediction, but their routine implementation requires further validation using independent external datasets, repeated resampling, and multi-instrument studies.

## Figures and Tables

**Table 1:** Selected hyperparameters for the hybrid CNN-based models.

PLS		
Parameter	ADF	CP
Latent variables	19	21
**Xgboost**		
**Parameter**	**ADF**	**CP**
min_child_weight	2	7
max_depth	10	10
learning_rate	0.25	0.15
gamma	0.3	0.4
colsample_bytree	1.0	1.0
n_estimators	100	100
subsample	1.0	1.0
objective	reg:squarederror	reg:squarederror
reg_alpha (L1)	0	0
reg_lambda (L2)	1	1
random_state	42	42

Note: For CNN+PLS, the reported number of components refers to PLS components selected from CNN-derived latent feature vectors, not from the original spectral wavelength variables. Component selection was performed by cross-validation within the training set. Parameters not explicitly tuned during grid search were kept at the default values of the XGBoost implementation.

**Table 2:** Overall calibration statistics for CP and ADF prediction using the pooled multi-product dataset.

Parameter	Model	TRN		
		R^2^_TRN_	MAE	RMSE
CP	GCM	0.97	0.66	0.88
	GCP	0.98	0.63	0.90
	CNN	0.99	0.40	0.54
	CNN+PLS	0.99	0.38	0.51
	CNN+XGBoost	0.99	0.40	0.53
ADF	GCM	0.99	0.99	1.54
	GCP	0.99	0.95	1.49
	CNN	0.99	0.89	1.21
	CNN+PLS	1.00	0.87	1.18
	CNN+XGBoost	0.99	1.43	1.52

**Table 3:** Validation statistics for CP and ADF prediction using the pooled multi-product dataset.

Parameter	Model	TST						
		R^2^_TST_	MAE	RMSEP	Bias	Slope	SEP	RPD_TST_
CP	GCM	0.98	0.55	0.73	+0.09	0.96	0.72	2.22
	GCP	0.98	0.56	0.77	−0.11	0.95	0.76	2.11
	CNN	0.98	0.47	0.62	+0.05	0.98	0.62	2.58
	CNN+PLS	0.98	0.45	0.60	−0.04	0.99	0.60	2.67
	CNN+XGBoost	0.98	0.45	0.60	+0.05	0.98	0.60	2.67
ADF	GCM	0.99	1.05	1.44	+0.14	0.97	1.43	3.36
	GCP	0.99	1.03	1.42	+0.13	0.97	1.41	3.40
	CNN	0.99	1.02	1.38	−0.08	0.99	1.38	3.48
	CNN+PLS	0.99	1.01	1.39	−0.07	0.99	1.39	3.45
	CNN+XGBoost	0.99	1.62	1.78	+0.28	0.95	1.76	2.73

Note: CP, crude protein; ADF, acid detergent fiber; TRN, training set; TST, testing set; R^2^_TRN,_ coefficient of determination for training; R^2^_TST_, coefficient of determination for testing; MAE, mean absolute error; RMSEP, root mean square error of prediction. GCM, Global PLSR model using the pooled multi-product database; GCP, product-specific PLSR model; Bias, mean prediction bias; Slope, slope of the regression between predicted and reference values; SEP, standard error of prediction; RPD_TST_, ratio of performance to deviation for the testing set.

**Table 4:** Product-wise calibration statistics for ADF prediction using product-specific PLSR and CNN-based models on the training dataset.

Model	Product	TRN		
		R^2^	MAE	RMSE
Global PLSR (GCM)	Hay	0.88	1.60	2.00
	Corn	0.84	0.59	1.02
	CSL	0.86	1.09	1.49
	Wheat	0.88	0.68	0.90
	TMR	0.91	1.41	1.93
	SGS	0.88	1.91	2.38
	Oat	0.89	0.91	1.08
Product-specific PLSR (GCP)	Hay	0.94	1.28	1.60
	Corn	0.96	0.38	0.68
	CSL	0.96	0.78	1.08
	Wheat	0.96	0.50	0.68
	TMR	0.98	1.10	1.54
	SGS	0.93	1.62	2.02
	Oat	0.98	0.64	0.77
CNN	Hay	0.94	1.02	1.28
	Corn	0.79	0.5	0.98
	CSL	0.89	0.96	1.32
	Wheat	0.38	0.45	0.61
	TMR	0.99	1.12	1.60
	SGS	0.97	1.52	1.82
	Oat	0.72	1.14	1.32
CNN+PLS	Hay	0.93	1.14	1.40
	Corn	0.78	0.54	1.09
	CSL	0.88	1.02	1.42
	Wheat	0.35	0.48	0.65
	TMR	0.99	1.26	1.77
	SGS	0.96	1.67	1.94
	Oat	0.70	1.27	1.42
CNN+XGBoost	Hay	0.91	1.12	1.42
	Corn	0.93	0.38	0.53
	CSL	0.87	0.92	1.28
	Wheat	0.54	0.36	0.49
	TMR	0.96	1.78	2.44
	SGS	0.90	1.85	2.55
	Oat	0.78	0.78	1.05

**Table 5:** Product-wise independent internal hold-out validation statistics for ADF prediction using product-specific PLSR and CNN-based models on the test dataset.

Model	Product	TST						
		R^2^_TST_	MAE	RMSEP	Bias	Slope	SEP	RPD_TST_
Global PLSR (GCM)	Hay	0.86	1.36	1.66	−0.25	0.91	1.64	2.74
	Corn	0.82	0.38	0.67	−0.08	0.92	0.67	2.99
	CSL	0.82	1.00	1.36	+0.21	0.92	1.34	2.61
	Wheat	0.60	0.45	0.61	+0.08	0.91	0.60	3.67
	TMR	0.90	1.45	1.96	−0.31	0.93	1.94	2.58
	SGS	0.89	1.88	2.29	+0.27	0.93	2.27	2.42
	Oat	0.78	2.77	3.28	−0.41	0.92	3.25	1.48
Product-specific PLSR (GCP)	Hay	0.93	1.06	1.30	+0.11	0.95	1.30	3.46
	Corn	0.96	0.24	0.43	−0.04	0.96	0.43	4.65
	CSL	0.93	0.69	0.96	+0.07	0.96	0.96	3.65
	Wheat	0.69	0.32	0.44	−0.04	0.93	0.44	5.00
	TMR	0.98	1.10	1.53	+0.11	0.96	1.53	3.27
	SGS	0.95	1.57	1.91	−0.14	0.96	1.90	2.89
	Oat	0.88	2.35	2.78	+0.28	0.96	2.77	1.73
CNN	Hay	0.92	1.09	1.40	−0.10	0.98	1.40	3.21
	Corn	0.91	0.47	0.62	+0.08	0.94	1.09	1.83
	CSL	0.88	0.86	1.22	−0.07	0.96	1.42	2.46
	Wheat	0.39	0.44	0.59	+0.04	0.89	0.65	3.38
	TMR	0.97	1.46	2.05	−0.11	0.98	1.77	2.82
	SGS	0.87	2.00	2.84	+0.13	0.96	1.94	2.84
	Oat	0.65	1.10	1.40	−0.10	0.92	1.42	3.38
CNN+PLS	Hay	0.91	1.14	1.40	+0.07	0.99	1.40	3.21
	Corn	0.71	0.54	1.09	−0.04	0.96	1.09	1.83
	CSL	0.84	1.02	1.42	+0.05	0.98	1.42	2.46
	Wheat	0.28	0.48	0.65	−0.02	0.89	0.65	3.38
	TMR	0.98	1.26	1.77	+0.09	0.97	1.77	2.82
	SGS	0.94	1.67	1.94	−0.07	0.98	1.94	2.84
	Oat	0.64	1.27	1.42	+0.08	0.92	1.42	3.38
CNN+XGBoost	Hay	0.89	1.30	1.61	+0.27	0.93	1.59	2.83
	Corn	0.91	0.43	0.6	+0.10	0.97	0.59	3.39
	CSL	0.84	1.01	1.40	−0.20	0.94	1.39	2.52
	Wheat	0.49	0.39	0.54	+0.08	0.89	0.53	4.15
	TMR	0.94	2.01	2.76	+0.42	0.92	2.73	1.83
	SGS	0.88	2.09	2.79	+0.38	0.93	2.76	1.99
	Oat	0.75	0.91	1.20	−0.19	0.94	1.18	4.07

**Table 6:** Product-wise calibration statistics for CP prediction using product-specific PLSR and CNN-based models on the training dataset.

Model	Product	TRN		
		R^2^_TRN_	MAE	RMSE
Global PLSR (GCM)	Hay	0.91	1.14	1.42
	Corn	0.87	0.68	0.88
	CSL	0.88	0.82	1.04
	Wheat	0.91	0.66	0.86
	TMR	0.93	0.69	0.89
	SGS	0.92	1.14	1.35
	Oat	0.85	0.98	1.12
Product-specific PLSR (GCP)	Hay	0.97	0.90	1.14
	Corn	0.99	0.45	0.59
	CSL	0.96	0.58	0.75
	Wheat	0.99	0.49	0.62
	TMR	1.00	0.53	0.67
	SGS	0.99	0.92	1.06
	Oat	0.93	0.73	0.80
CNN	Hay	0.97	0.55	0.70
	Corn	0.98	0.27	0.36
	CSL	0.96	0.36	0.46
	Wheat	0.98	0.30	0.38
	TMR	1.00	0.32	0.41
	SGS	0.99	0.56	0.65
	Oat	0.95	0.44	0.49
CNN+PLS	Hay	0.97	0.61	0.77
	Corn	0.98	0.29	0.37
	CSL	0.96	0.38	0.49
	Wheat	0.98	0.32	0.41
	TMR	1.00	0.35	0.44
	SGS	0.99	0.60	0.69
	Oat	0.94	0.47	0.51
CNN+XGBoost	Hay	0.96	0.80	1.00
	Corn	0.98	0.31	0.4
	CSL	0.94	0.43	0.55
	Wheat	0.93	0.49	0.61
	TMR	0.99	0.61	0.78
	SGS	0.97	0.89	0.99
	Oat	0.85	0.61	0.73

**Table 7:** Product-wise independent internal hold-out validation statistics for CP prediction using product-specific PLSR and CNN-based models on the test dataset.

Model	Product	TST						
		R^2^_TST_	MAE	RMSEP	Bias	Slope	SEP	RPD_TST_
Global PLSR (GCM)	Hay	0.90	0.82	1.04	+0.14	0.91	1.03	3.11
	Corn	0.86	0.41	0.52	−0.07	0.92	0.52	2.88
	CSL	0.87	0.50	0.64	−0.09	0.92	0.63	3.17
	Wheat	0.90	0.44	0.55	+0.07	0.91	0.55	3.27
	TMR	0.92	0.50	0.63	−0.09	0.93	0.63	3.97
	SGS	0.91	0.88	1.04	+0.15	0.92	1.03	3.40
	Oat	0.79	0.97	1.13	−0.17	0.91	1.12	1.96
Product-specific PLSR (GCP)	Hay	0.96	0.64	0.81	+0.07	0.96	0.81	3.95
	Corn	0.98	0.27	0.34	−0.03	0.97	0.34	4.41
	CSL	0.95	0.35	0.45	+0.04	0.96	0.45	4.44
	Wheat	0.98	0.3	0.38	−0.03	0.97	0.38	4.74
	TMR	0.99	0.36	0.45	+0.04	0.97	0.45	5.56
	SGS	0.98	0.65	0.75	−0.06	0.96	0.75	4.67
	Oat	0.88	0.74	0.80	+0.09	0.95	0.80	2.75
CNN	Hay	0.96	0.64	0.82	+0.06	0.97	0.82	3.90
	Corn	0.97	0.29	0.37	−0.02	0.98	0.37	4.05
	CSL	0.96	0.33	0.45	+0.03	0.98	0.45	4.44
	Wheat	0.92	0.50	0.62	−0.05	0.96	0.62	2.90
	TMR	0.98	0.47	0.64	+0.04	0.98	0.64	3.91
	SGS	0.98	0.58	0.71	−0.04	0.99	0.71	4.93
	Oat	0.65	0.78	1.06	+0.13	0.90	1.05	2.10
CNN+PLS	Hay	0.96	0.61	0.77	+0.04	0.99	0.77	4.16
	Corn	0.97	0.29	0.37	+0.02	0.99	0.37	4.05
	CSL	0.95	0.38	0.49	+0.02	0.98	0.49	4.08
	Wheat	0.97	0.32	0.41	−0.02	0.99	0.41	4.39
	TMR	0.99	0.35	0.44	+0.02	0.99	0.44	5.68
	SGS	0.98	0.60	0.69	−0.03	0.99	0.69	5.07
	Oat	0.92	0.47	0.51	+0.03	0.98	0.51	4.31
CNN+XGBoost	Hay	0.94	0.8	1.00	+0.16	0.94	0.99	3.23
	Corn	0.96	0.35	0.45	+0.07	0.96	0.45	3.33
	CSL	0.94	0.43	0.55	−0.09	0.95	0.54	3.70
	Wheat	0.93	0.49	0.61	+0.10	0.93	0.60	3.00
	TMR	0.97	0.61	0.78	+0.13	0.94	0.77	3.25
	SGS	0.96	0.89	0.99	+0.17	0.94	0.98	3.57
	Oat	0.83	0.61	0.73	+0.12	0.92	0.72	3.06

## Data Availability

The original spectral and reference database used in this study was derived from a previously published dataset and is subject to data-use restrictions. Therefore, the complete raw database and full model-training code are not publicly deposited. To improve transparency and reproducibility, the train/hold-out test indices for the CP and ADF modelling datasets are provided in Supplementary Table S2. The R script documenting the spectral preprocessing pipeline, including SNVD followed by Savitzky–Golay smoothing and first-order derivative transformation, is provided as Supplementary Code S1. Additional information may be made available from the corresponding authors upon reasonable request, subject to data-use restrictions and institutional approval.

## Supplementary Materials

The following supporting information can be downloaded at: https://www.mdpi.com/article/10.3390/ani16111676/s1. Code S1: R script documenting the spectral preprocessing pipeline, including standard normal variate followed by detrending (SNVD), Savitzky–Golay smoothing, first-order derivative transformation, and edge wavelength handling; Table S1: Product-wise numbers of samples assigned to the training and independent internal hold-out test sets for CP and ADF modelling datasets; Table S2: Train and hold-out test sample indices for the CP and ADF modelling datasets; Figure S1: Raw near-infrared absorbance spectra of feed samples displayed separately by product category; Figure S2: Mean raw near-infrared absorbance spectra by product category.
