# Supplementary material for: Convolutional Neural Network-Based Models for Near-Infrared Prediction of Nutritional Quality in Multi-Product Animal Feeds
Source: Animals (Basel). 2026 May 30;16(11):1676. doi: 10.3390/ani16111676 (PMC13255911; doi:10.3390/ani16111676)
Supplement: Supplementary file 1 [file animals-16-01676-s001.zip › Supplementary Code S1 R script documenting the spectral preprocessing pipeline.pdf]

```r
# Supplementary Code S1. R script documenting the spectral preprocessing pipeline, including standard normal variate followed by detrending (SNVD), Savitzky – Golay smoothing, first-order derivative transformation, and edge wavelength handling
library(prospectr)

# X.raw: spectral matrix with samples in rows and wavelengths in columns.
# Original wavelength range: 1100 – 2498 nm at 2 nm intervals.
# The same preprocessing pipeline was applied to all models.

# 1. Standard normal variate followed by detrending
X.snvd <- standardNormalVariate(X.raw)
X.snvd <- detrend(X.snvd)

# 2. Savitzky – Golay smoothing and first derivative
# p = 2: polynomial order
# w = 7: window size
# m = 1: first derivative
X.snvd.sg <- savitzkyGolay(X = X.snvd, p = 2, w = 7, m = 1)

# 3. Edge handling
# No padding or extrapolation was applied.
# With w = 7, three wavelength variables at each spectral edge are removed.
# Original range: 1100 – 2498 nm, 700 variables.
# Final range after SG: 1106 – 2492 nm, 694 variables.
```
