# Supplementary material for: Convolutional Neural Network-Based Models for Near-Infrared Prediction of Nutritional Quality in Multi-Product Animal Feeds
Source: Animals (Basel). 2026 May 30;16(11):1676. doi: 10.3390/ani16111676 (PMC13255911; doi:10.3390/ani16111676)
Supplement: Supplementary file 1 [file animals-16-01676-s001.zip › Supplementary Figure S1 Raw near-infrared absorbance spectra of feed samples displayed separately by product category.pdf]

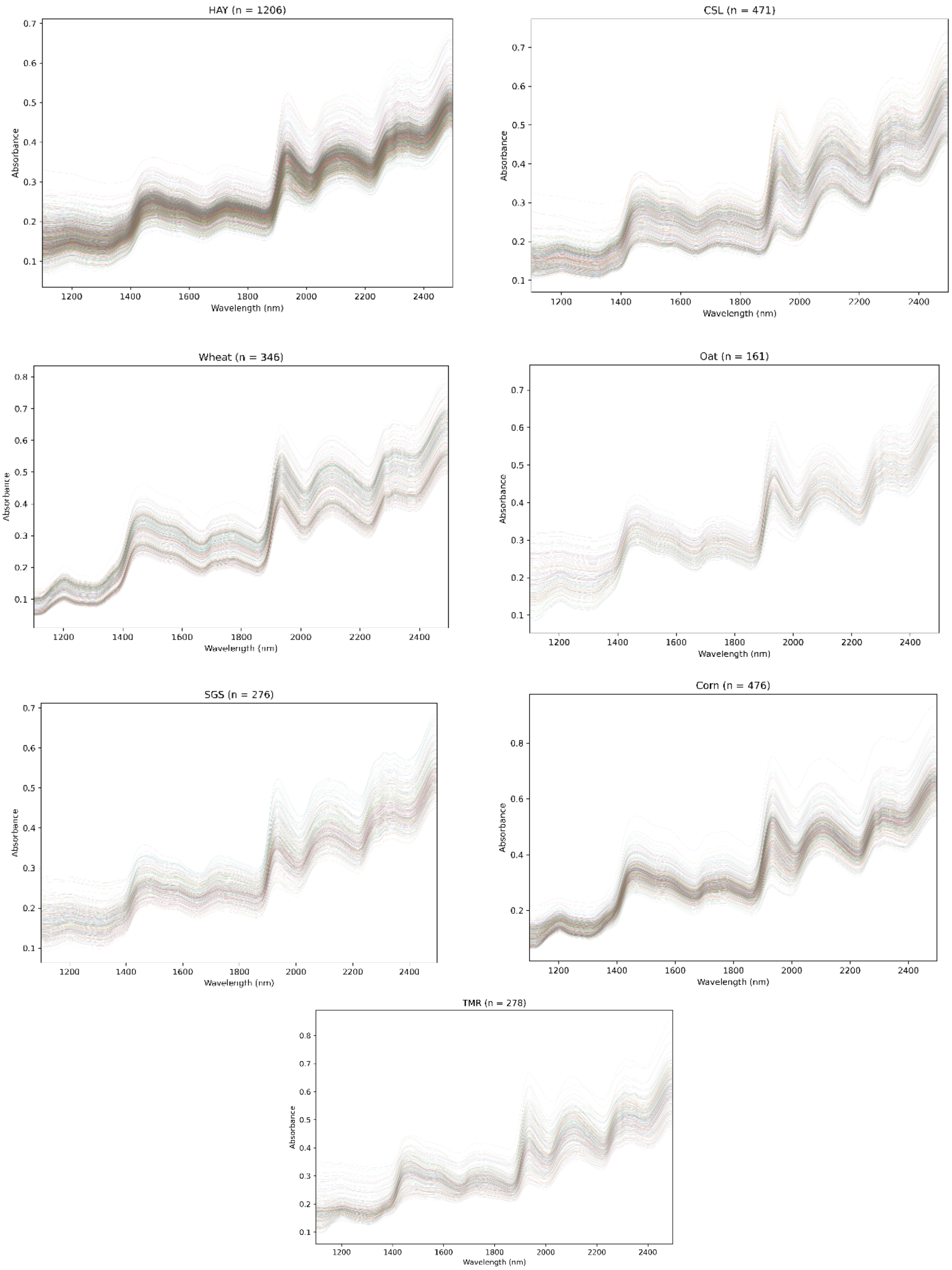

Supplementary Figure S1. Raw near-infrared spectra of all available unique feed samples before spectral preprocessing. The figure was generated using the combined CP modelling dataset (training + testing), which contained 3214 unique samples and included all samples used for ADF modelling. Spectra were recorded over the wavelength range of **1100–2498 nm**. The x-axis represents **Wavelength (nm)** and the y-axis represents **Absorbance**.
