# Supplementary material for: Convolutional Neural Network-Based Models for Near-Infrared Prediction of Nutritional Quality in Multi-Product Animal Feeds
Source: Animals (Basel). 2026 May 30;16(11):1676. doi: 10.3390/ani16111676 (PMC13255911; doi:10.3390/ani16111676)
Supplement: Supplementary file 1 [file animals-16-01676-s001.zip › Supplementary Figure S2 Mean raw near-infrared absorbance spectra by product category.pdf]

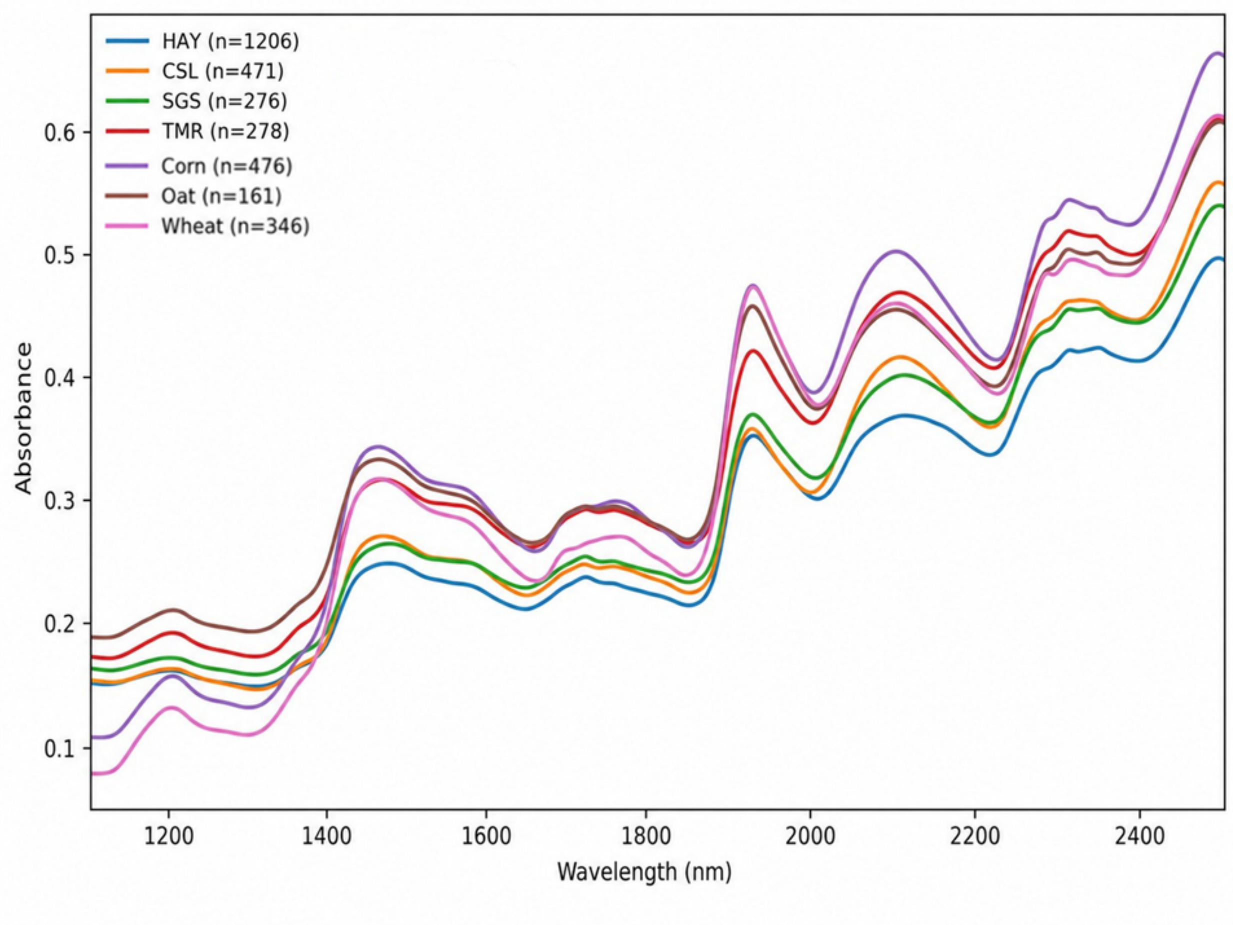

Supplementary Figure S2. Mean raw near-infrared spectra by product category before spectral preprocessing. Mean spectra are shown for HAY, CSL, SGS, TMR, Corn, Oat, and Wheat. The number of unique samples used for each category is indicated in the legend. Spectra were recorded over the wavelength range of **1100–2498 nm**. The x-axis represents **Wavelength (nm)** and the y-axis represents **Absorbance**.
