# Supplementary material for: Convolutional Neural Network-Based Models for Near-Infrared Prediction of Nutritional Quality in Multi-Product Animal Feeds
Source: Animals (Basel). 2026 May 30;16(11):1676. doi: 10.3390/ani16111676 (PMC13255911; doi:10.3390/ani16111676)
Supplement: Supplementary file 1 [file animals-16-01676-s001.zip › Table S1 Product-wise numbers of samples assigned to the training and independent internal hold-out test sets for CP and ADF modelling datasets.pdf]

Table S1: Product-wise numbers of samples assigned to the training and independent internal hold-out test sets for CP and ADF modelling datasets

Product	CP total	CP training	CP testing	ADF total	ADF training	ADF testing
HAY	1206	1085	121	1206	1085	121
CSL	471	424	47	471	424	47
SGS	276	248	28	276	248	28
TMR	278	250	28	275	248	27
Corn	476	428	48	441	397	44
Oat	161	145	16	161	145	16
Wheat	346	311	35	321	289	32
**Total**	**3214**	**2891**	**323**	**3151**	**2836**	**315**
